# Study on Genotyping Polymorphism and Sequencing of N-Acetyltransferase 2 (NAT2) among Al-Ahsa Population

**DOI:** 10.1155/2020/8765347

**Published:** 2020-06-15

**Authors:** Mohammad Abu Zahra, Mahmoud Kandeel, Sara A. Aldossary, Abdulla Al-Taher

**Affiliations:** ^1^Department of Biomedical Sciences, College of Veterinary Medicine, King Faisal University, 31982 Al-Ahsa, Saudi Arabia; ^2^Department of Pharmacology, Faculty of Veterinary Medicine, Kafrelsheikh University, 33516 Kafrelsheikh, Egypt; ^3^Department of Pharmaceutical Sciences, College of Clinical Pharmacy, King Faisal University, 31982 Al-Ahsa, Saudi Arabia

## Abstract

One of the well-studied phase II drug metabolizing enzymes is N-acetyltransferase 2 (*NAT2*) which has an essential role in the detoxification and metabolism of several environmental toxicants and many therapeutic drugs like isoniazid (antituberculosis, TB) and antimicrobial sulfonamides. According to the variability in the acetylation rate among different ethnic groups, individuals could be classified into slow, intermediate, and fast acetylators; these variabilities in the acetylation rate are a result of single nucleotide polymorphisms (SNPs) in the coding sequence of *NAT2*. The variety of *NAT2* acetylation status is associated with some diseases such as bladder cancer, colorectal cancer, rheumatoid arthritis, and diabetes mellitus. The main objectives of this research are to describe the genetic profile of *NAT2* gene among the people of the Al-Ahsa region, to detect the significant SNPs of this gene, to determine the frequency of major *NAT2* alleles and genotypes, and then categorize them into fast, intermediate, and slow acetylators. Blood samples were randomly collected from 96 unrelated people from Al-Ahsa population, followed by DNA extraction then amplifying the *NAT2* gene by polymerase chain reaction (PCR); finally, functional *NAT2* gene (exon 2) was sequenced using the Sanger sequencing method. The well-known seven genetic variants of *NAT2* gene are 191G>A, 282C>T, 341T>C, 481C>T, 590G>A, 803A>G, and 857G>A were detected with allele frequencies 1%, 35.4%, 42.7%, 41.1%, 29.2%, 51%, and 5.7%, respectively. The most common *NAT2* genetic variant among Al-Ahsa population was 803A>G with a high frequency 0.510 (95% confidence interval 0.44-0.581) followed by 341T>C 0.427 (95% confidence interval 0.357-0.497). The most frequent two haplotypes of *NAT2* were *NAT2*∗*6C* (25.00%) and *NAT2*∗*5A* (22.92%) which were classified as a slow acetylators. According to trimodal distribution of acetylation activity, the predicted phenotype of Al-Ahsa population was found to be 5.21% rapid acetylators, 34.38% intermediate acetylators, and 60.42% were slow acetylators. In addition, this study found four novel haplotypes *NAT2*∗*5*TB, *NAT2*∗*5AB*, *NAT2*∗*5ZA*, and *NAT2*∗*6*W which were slow acetylators. This study revealed a high frequency of the *NAT2* gene with slow acetylators (60.42%) in Al-Ahsa population, which might alter the drug's efficacy and vulnerability to some diseases.

## 1. Introduction

Human *NAT2* gene has a key role in the metabolism of hydrazines, arylamines, several environmental toxicants, and therapeutic drugs like isoniazid and the antimicrobial sulfonamides by the N-acetylation of aromatic amines as well as the O-acetylation of carcinogenic heterocyclic amines [1]. This gene is present on the chromosome number 8, at Cytoband p22, and has a coding region of exon 2 with 870 bp which encodes 290 amino acids, and it is mainly expressed in the liver and small intestine [2]. The interest of studying the variability in N-acetylation status of *NAT2* has been increased among ethnic groups due to the importance of *NAT2* polymorphism as biomarkers to evaluate the efficacy of therapy and toxicity during treatment or minimization of adverse drug reactions (ADR) [3].

There is a relationship between *NAT2* acetylation status with some diseases like bladder cancer, colorectal cancer, rheumatoid arthritis, and diabetes [3]. According to the variability in the acetylation rate among different ethnic groups, individuals are classified based on acetylation phenotypes as slow, intermediate, and rapid acetylators. This variability in the acetylation rate is a result of SNPs in the coding sequence of *NAT2* [4]. The plasma drug concentration in slow acetylators remains higher than that in the rapid acetylators which may be correlated with adverse drug reactions (ADR) [5]. The most commonly found seven SNPs are *NAT2*∗*5*, *NAT2*∗*6*, *NAT2*∗*7*, *NAT2*∗*14*, *NAT2*∗*11*, *NAT2*∗*12*, and *NAT2*∗*13* [6]. The wild-type *NAT2*∗*4* allele does not have any nucleotide substitutions and is associated with fast acetylator phenotype [7].

Ample literature studies show interethnic variation in acetylation. In addition, many studies showed a great variation between Arabs and non-Arabs in drugs and environmental chemical acetylation. Arabs, like others, are not a single race, and possible variation in drug metabolism is still existing. This could be due to the presence of different SNPs. Al-Ahsa is one of the most populated oasis since ancient times in the Arabian peninsula. Several ethnic groups migrated and settled at Al-Ahsa and merged to be the present inhabitants of Al-Ahsa. This may have made Al-Ahsa characterized by a mixed population structure, genetic makeup, and a host of special diseases such as sickle cell anemia. Because of the above, Al-Ahsa may be a promising place to study the genetic makeup of drug metabolizing enzymes and its role in drug response. Since there was no previous study of *NAT2* (SNPs) in Al-Ahsa, so, this study was proposed. Hence, the present research is aimed at sequencing the *NAT2* gene and identifying the gene polymorphisms, which may regulate the interethnic and interindividual phenotypes in drug's activity and toxicity.

## 2. Materials and Methods

### 2.1. Sample Collection

A total of 96 blood samples were randomly collected from unrelated individuals from different dispensaries of Al-Ahsa, Saudi Arabia. A volume of 3 ml of venous blood samples were drawn and collected in EDTA tubes. Blood samples were placed in ice bags and then transported to the King Faisal University (KFU) laboratory where they were refrigerated at 4 (°C) for DNA extraction.

Volunteers have been informed about the nature and the aim of the study, and they were asked to sign a consent before collection of samples. This work was approved by the Research Ethics Committee (REC) at King Faisal University (REC REF Number: KFU-REC/2017-11-02) and clinical ethical committee at King Fahad Hospital, Hufof (KFHH RCA NO: 07/09/39).

### 2.2. DNA Extraction and Quantification

The Wizard® Genomic DNA purification kit (Promega, Madison, USA) was used for extraction of genomic DNA from the white blood cells in each sample. The extracted gDNA is quantified by using a Nanodrop Spectrophotometer instrument (Synergy™ Mx Monochromator-Based Multi-Mode Microplate Reader, Bio-Tek, USA) and makes up the final concentration of 100 ng/*μ*l by nuclease-free water.

### 2.3. Primer Design and *NAT2* Gene Amplification by Polymerase Chain Reaction (PCR)

The amplification of the *NAT2* gene (exon 2) was carried out with a thermal cycler (C1000, Bio-Rad, Singapore). The PCR primers were designed according to the *NAT2* sequence obtained from the National Center For Biotechnological Information (NCBI) and purchased from Macrogen, Korea. The *NAT2*-specific primers were designed to amplify the exon 2 region of the *NAT2* gene, forward primer 5′GGGATCATGGACATTGAAGCA3′ and reverse primer 5′ATGTTTTCTAGCATGAATCACTCTG (Macrogen, Seoul, Korea). The covering of the functional part of exon 2 (870 bp) and its boundaries has a total length of 1150 bp. The PCR mixture consists of 100 ng of genomic DNA, 2X Mastermix (2X Green GoTaq® Reaction Buffer (pH 8.5), 400 *μ*M dATP, 400 *μ*M dGTP, 400 *μ*M dCTP, 400 *μ*M dTTP, and 3 mM MgCl2), and 10 picomoles of forward and reverse primers to make up the final reaction volume 50 *μ*l. The PCR program was set as initial denaturation at 95°C for 3 minutes, 35 cycles were performed consisting of a denaturation step at 95°C for 30 seconds, an annealing step at 60°C for 30 seconds, and an elongation step at 72°C for 1 minute, completed with a final cycle of elongation at 72°C for 5 minutes.

### 2.4. *NAT2* DNA Sequencing

The 1150 bp amplicons of the *NAT2* genes were sent to Macrogen company (Macrogen, Seoul, Korea**)** for sequencing. The detection of *NAT2* variations was carried out by using a bidirectional high-throughput capillary sequencing based on Sanger's dideoxy chain-termination DNA sequencing method.

### 2.5. Sequence Data Analyses

The raw sequencing data were analyzed using a Mutation Surveyor® program (Pennsylvania, USA). It is a powerful, sensitive software and an accurate DNA sequencing analysis tool for Sanger sequencing files produced by Applied Biosystems Genetic Analyzers. For alignment, comparison, and screening for different SNPs, the variations of sample DNA sequence traces are compared to reference wild-type sequence from GenBank database. Each sequence was submitted to the program and compared with the *NAT2* reference sequence. Moreover, the sequences of samples were evaluated for chromatogram peaks and overlapping bases to make consensus sequences. The homozygous and heterozygous SNPs cannot be differentiated by the software, so it has to look for each DNA sequence by naked eyes. Depending on the peak of the chromatogram, if there is a single clear and normal peak, this reflects that we have homozygous SNP, but if the peak was short with the presence of double peaks instead of one peak with different colors, that is an indication for heterozygous SNP.

### 2.6. Acetylator Phenotype Classification

Based on the level of *NAT2* enzymatic activity, populations are divided into three *NAT2* acetylator phenotypes: slow, intermediate, and rapid [4]. Hence, the predicted phenotypes in the current study were predicted from genotypes as three types of acetylators. A slow acetylator was predicted if the genotype was comprised of 2 slow alleles, a rapid acetylator genotype would consist of 2 rapid alleles, and an intermediate acetylator would be predicted if its genotype contained one slow and another rapid acetylator allele. The alleles considered rapid were the wild (*NAT2*∗*4*) ones and those containing polymorphisms of 282C>T (*NAT2*∗*13*), 481C>T (*NAT2*∗*11*), and 803A>G (*NAT2*∗*12*), while all other alleles 341T>C (*NAT2*∗*5*), 590G>A (*NAT2*∗*6*), 857G>A (*NAT2*∗*7*), and 191G>A (*NAT2*∗*14*) were considered slow acetylators [8]. Online software *NAT2*PRED program (http://nat2pred.rit.albany.edu/) was used for inferring the human *NAT2* acetylator phenotype that uses a combination of SNPs found in the *NAT2* gene positions 282, 341, 481, 590, 803, and 857 [9].

### 2.7. Statistical Analysis

The chi-squared (*χ*^2^) test was performed on observed and expected genotype frequencies to find out whether the genotype distribution was in Hardy–Weinberg equilibrium and to compare frequencies of genotypes and alleles in the group. The probability level of *p* < 0.05 was considered the cut-off value for significance.

## 3. Results

### 3.1. Genomic DNA Extraction

The extracted genomic DNA was run on 1.5% agarose gel; then, the DNA bands were confirmed and visualized by Gel Doc™ and ChemiDoc™ Systems, Bio-Rad, USA. Gel electrophoresis of genomic DNA was done to confirm the presence of human genomic DNA in each sample.

### 3.2. *NAT2* Amplification

**Figure 1:**
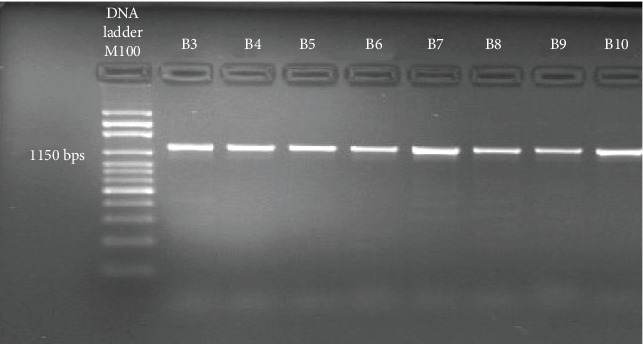
The PCR gel picture representing the 1150 bp of *NAT2* amplified fragment.

This electrophoresis gel photograph shows the amplified fragment of the *NAT2* gene. The band of 1150 bp was confirmed by using DNA ladder as shown in Figure 1.

### 3.3. *NAT2* Sequence Analysis

The commonly known seven SNPs detected in our study were 191G>A, 282C>T, 341T>C, 481C>T, 590G>A, 803A>G, and 857G>A and their frequencies are represented in Table 1. It has been noted that heterozygote SNPs are more common compared to homozygote SNPs (198 versus 99, respectively) as shown in Table 2.

### 3.4. Allele Frequency

The frequencies of these major *NAT2* SNPs in all of the 96 individuals are represented in Table 1. This study found that *NAT2*∗*12*, 803A>G, was the most frequent genetic variant (51% of alleles) among Al-Ahsa population, whereas the genetic variant of *NAT2*∗*14*, 191G>A, was the least with allele frequency of only 1% of alleles. The allele not harboring any mutation was considered wild-type *NAT2*∗*4*, fast acetylator, which was present only in 2 cases of the 96 samples. So, the frequency of *NAT2*∗*4/*∗*4* was 2%.

### 3.5. Genotype Frequency

The frequencies of *NAT2* genotype obtained from all of subjects are publicized in Table 2. The most frequent observed heterozygote was *NAT2*∗*13* 282C>T (43.7%) followed by *NAT2*∗*5* 341T>C (39.5%) and *NAT2*∗*12* 803A>G (39.5%) among Al-Ahsa volunteers. The lowest frequency of observed heterozygote genotypes was *NAT2*∗*14* 191G>A with a frequency of 2%. In homozygote, the *NAT2*∗*12* 803A>G (31.2%) genotype was the most common one but the lowest homozygote among them was *NAT2*∗*6* 590G>A (12.5%), and there was no any homozygote genotypes of *NAT2*∗*7* 587G>A and *NAT2*∗*14* 191G>A in the present study.

The Hardy–Weinberg equilibrium test was done by using the chi-squared (*χ*^2^) test. Five of the seven most common SNPs were within Hardy-Weinberg equilibrium while two of them are not shown in Table 3.

### 3.6. Linkage Disequilibrium

**Figure 2:**
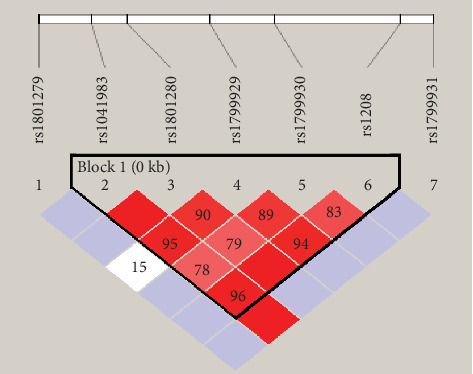
Linkage disequilibrium (LD) of *NAT2* genetic variants found among Al-Ahsa population samples. The LD was carried out using Haploview software. The upper panel shows the location of 7 variants in the NAT2 gene, and the lower panel shows the output of Haploview. The red squares represent a strong LD, and the white square represents a weak LD. The blue square indicates that there is no LD.

The linkage disequilibrium (LD) analysis was done using Haploview 4.2 software to indicate the heterogeneity of Al-Ahsa population and the possibility of recombination among their *NAT2* SNPs. As shown in Figure 2, the seven *NAT2* variants, 191G>A, 282C>T, 341T>C, 481C>T, 590G>A, 803A>G, and 857G>A were applied to Haploview software. The LD for each pair of genetic variants was measured using ∣D′∣ and correlation coefficient (*r*^2^ > 0.8). A haplotype block was found in the following SNP positions 282C>T, 341T>C, 481C>T, 590G>A, and 803A>G in Al-Ahsa population samples which is identified as strong LD as represented in Figure 2.

### 3.7. Acetylation Phenotype

According to the different *NAT2* polymorphisms, it will characterize the phenotypes into three different acetylators known as slow, intermediate, and fast [4], hence the predicted phenotypes in our study based on the various *NAT2* haplotypes (Table 4). Consequently, our study revealed that the genotype frequency (predicted phenotype) of fast, intermediate, and slow acetylators was 5.21%, 34.38%, and 60.42%, respectively (Table 4).

Many researchers who published work on *NAT2*, subdivided *NAT2*∗ phenotypes into either “slow” or “rapid” acetylators which is described as a bimodal pattern. However, the third type of acetylators, namely, “intermediate” is also mentioned in most of the *NAT2* studies which have been called as a trimodal pattern. Both of them, bi- and trimodal acetylation classifications, have been found in the literature [10, 11].

### 3.8. Haplotype Frequency

The detected haplotypes of the *NAT2* gene in the current study among Al-Ahsa population have been resulted from various SNPs existing together in one individual as explained in the NAT database nomenclature official website. In the NAT2 gene, the predicted acetylation phenotypes and hence the metabolic capacity of this enzyme is determined by the haplotype structure for each individual. In the current study, we found in total 20 different haplotypes in Al-Ahsa population as reported in Table 5.

The most frequent two haplotypes in the present study were *NAT2*∗*6C* and *NAT2*∗*5A* with a frequency of 25% and 22.9%, respectively, followed by *NAT2*∗*5B* and *NAT2*∗*5U* with a frequency of 11.5% and 12.5% correspondingly. That indicates most of examined samples of Al-Ahsa population were slow acetylators according to the bimodal distribution pattern with a proportion of 94.8% of slow acetylator phenotype and 5.21% of fast acetylator phenotype.

Our genotyping results detected four novel haplotypes which are not published yet on the *NAT* database nomenclature official website (http://nat.mbg.duth.gr/human%20nat2%20alleles_2013.htm). The present study applied an online software *NAT2*PRED program (http://nat2pred.rit.albany.edu/) in order to predict its acetylation phenotype. Thus, the predicted phenotype of these four new haplotypes is slow acetylators as shown in Table 5, and they represent 9.37% of the 96 samples. The new detected haplotypes were given an official symbol based on communication with the Chair of the *NAT* Committee as the following *NAT2*∗*5*TB, *NAT2*∗*5AB*, *NAT2*∗*5ZA*, and *NAT2*∗*6*W (Table 5), also see the attached supplementary file (available here).

### 3.9. Comparative Assessment of Al-Ahsa *NAT2* Allele Frequency with Other Populations

The *NAT2* genetic polymorphisms among the different ethnic groups and countries are common. Our work differentiates the frequencies of *NAT2* allele among Al-Ahsa population with other ethnicities as illustrated in Table 6.

## 4. Discussion

This study is the first to provide an extensive and comprehensive report on *NAT2* genotyping among Al-Ahsa population, in which all common polymorphisms of this specific gene have been thoroughly studied.

Unrelated samples were collected almost from each local district of Al-Ahsa to sufficiently cover most of the region, which represents a good sample distribution. The sample size of this study was relatively small which is correlated to a limitation of this research. Historically, it is undoubtedly perceived that Al-Ahsa inhabitants in the Eastern Province of Saudi Arabia have settled down the city thousands of years ago and genetic problems are very common among them due to the apparent prevalence of endogamy. As a result, Al-Ahsa has a special demographic composition and unique population structure [23, 24]. The acetylation rate has a great influence by the *NAT2* gene due to the presence of various SNPs, and it may affect the drug metabolism and susceptibility to some diseases such as cancer. In the current work, the *NAT2* gene whole exon 2 was sequenced from Al-Ahsa of Saudi population.

The frequency of slow *NAT2* alleles in the current study 341T>C (*NAT2*∗*5*), 590G>A (*NAT2*∗*6*), and 857G>A (*NAT2*∗*7*) were relatively similar to a previous Saudi study [12]. However, fast alleles 481C>T (*NAT2*∗*11*), 803A>G (*NAT2*∗*12*), and 282C>T (*NAT2*∗*13*) were not reported by Bu et al. compare to ours, which makes the current study more comprehensive.

The distribution of *NAT2* polymorphisms in Al-Ahsa population appears to be relatively different from other ethnically related Arab populations, especially in the United Arab Emirates and Oman [14, 15]. The wild-type allele (*NAT2*∗*4*) frequencies in Emiratis and Omanis are 0.13 and 0.18, respectively, compared with our study 0.295 [14, 15]. But it is slightly similar to Jordan and Egypt 0.23 and 0.22, respectively. Besides, the frequency of *NAT2*∗*4* allele in the current study is almost similar to Caucasians and different from South East Asians such as Japanese, Korean, and Chinese (Table 6).

The highest occurrence slow allele among Al-Ahsa population is 341T>C (*NAT2*∗*5*) with a frequency of 42.7%, where the least common slow allele is 191G>A (*NAT2*∗*14*) with a frequency of only 1%. The later SNP (191G>A) was not examined in the previous Saudi study [12]. Conversely, the (191G>A) SNP is absent in Omanis and European Caucasians, but it was reported among Emiratis with a significant frequency (9.96%), and it was also found among Jordanians with less frequency (0.7%). This polymorphism (191G>A) is considered African specific with a frequency of 9% [25]. Moreover, this may explain its lower frequency in the present study and/or absent in others. Besides, the dissimilarity in the distribution of this SNP (191G>A) could be due to a lower degree of admixture in Al-Ahsa population. It has been reported that slow acetylators *NAT2*∗*5B* and *NAT2*∗*5C* are associated with a low risk of acute lymphoblastic leukemia (ALL) development. On the contrary, *NAT2*∗*5D* is associated both with ALL and acute myeloblastic leukemia (AML) [26]. However, a Brazilian study demonstrated that *NAT2*∗*14*, *NAT2*∗*5A*, and *NAT2*∗*5C* are correlated with an increased susceptibility to ALL in a Brazilian population [27, 28]. Recently, a research group reviewed the published literature on the correlation between *NAT2* genetic variants and susceptibility to acute leukemia and it was reported that rs1801280 (341T>C (*NAT2*∗*5*)) contributes to the disease [29]. Having a high percentage of this *NAT2*∗*5A* polymorphism (23%) among Al-Ahsa community may make them vulnerable to acute leukemia, taking into account that the Eastern region is the second in the prevalence of leukemia in the Kingdom after Riyadh [30].

The observed frequency of slow acetylator allele *NAT2*∗*6* among Al-Ahsa community is 29% which is virtually consistent with other Arab countries (previous Saudi study 24%, Jordan 30%, Oman 30%, and Egypt 26%) and Caucasians 28%. However, Emirates showed a lesser occurrence of 10% of that SNP (Table 6). The association between different *NAT2* genotypes and risk for diabetes mellitus is controversial. Some groups showed no relationship between different *NAT2* SNPs or phenotypes and risk for diabetes mellitus [31–33]. However, other groups showed a relationship between different *NAT2* genotypes *NAT2*∗*6A*, *NAT2*∗*5A*, *NAT2*∗*14A*, *and NAT2*∗*7B* and risk for diabetes mellitus [34, 35]. The contemporary study revealed that the frequency of *NAT2*∗*7* (5.7%) was in line with Arab and Caucasians (Oman 4%, Jordan 3%, Egypt 3%, and Caucasians 2%), except for Emirates and Asians which have a slightly more frequent *NAT2*∗*7* allele 27% and 13%, respectively, (Table 6).

It has been abstracted that the fast acetylators are connected with colorectal cancer especially with people who consume huge amounts of well-done meat that considered a predominant source of the carcinogenic heterocyclic amines which metabolized by *NAT2* [36]. In addition, one thousands annually of Saudi patients out of 3 million people worldwide die of tuberculosis (TB) [37]. The infection rate of TB in the KSA remains high. This is reflected by the latest prevalence study which showed that TB range from 8.5% in the central region (Riyadh) to as high as 23.1% in Hail for locals and as high as 38% for non-Saudis in the Makkah region [38]. The most effective drug used for treatment of TB is isoniazid (INH) which is mainly metabolized by *NAT2* enzyme. The incidence of ADRs caused by INH is very high among slow acetylators, as Al-Ahsa population is mainly slow acetylators (60.42%) (Table 6). Therefore, the dosage regimen of INH should be adjusted in TB patients carrying slow genotypes to prevent drug-induced liver injuries (DILI) and to decrease the cost of managing adverse events [39]. *NAT2* genotypes are useful new biomarkers for predicting anti-TB DILI for TB patients. Accordingly, DNA-based diagnosis of TB patients before initiating treatment with anti-TB drugs may prove useful in achieving optimal treatment of individual TB patients.

In the present study, *NAT2*∗*5* prevalence is 42.7% and the *NAT2*∗*5* genotypes include (TT 37.5%, TC 39.6%, and CC 22.9%). Association between *NAT2*∗*5* and anemia in ovarian cancer patients under the treatment of cisplatin was reported. Strictly, anemia was more prevalent among patients with the heterozygous TC genotype (78.4%) compared with the homozygous wild-type [40]. Occasionally, heterozygous TC genotype, in our study, showed a 39.6% which reflects a high percentage of such SNP. So, it is recommended to do a *NAT2* genotyping test before cisplatin treatment to reduce its ADRs.

Endometriosis is defined as the growth of endometrium in the peritoneal cavity, outside the uterine or myometrium [41]. It frequently occur among women, at the rate of 6-10% [42]. The prevalence of endometriosis was found to be 11-14.3% in Saudi women [43]. The relationship between endometriosis and polymorphisms in *NAT2* gene was investigated in a UK population. The researchers reported that male controls account for 32% and unaffected women 33% consecutively. Therefore, they were found to be slow acetylators than the rate of 57% for women with stage III–IV endometriosis [42]. Interestingly, Fayez et al. found that the heterozygote genotype 590G A (*>NAT2*∗*6*) SNP may be linked with vulnerability to endometriosis and the homozygote genotype 590 AA allele may have a protective role in development of endometriosis in Iranian women [44]. Our study showed that the distribution of heterozygote genotype 590G>A (*NAT2*∗*6*) was found to be 33% of Al-Ahsa population. This high percentage of prevalence genotype among Al-Ahsa population might make them susceptible to be affected by endometriosis.

## 5. Conclusions

Our study revealed the high prevalence of slow acetylators of *NAT2* (60.42%) in the studied samples among Al-Ahsa population which might alter drug's efficacy and vulnerability to some diseases, like cancer. The result of this study will be helpful to limit the unwanted side effects of some medications and maximization of its benefits to the patients. Moreover, we found four novel haplotypes which were given a new official NAT symbols *NAT2*∗*5*TB, *NAT2*∗*5AB*, *NAT2*∗*5ZA*, and *NAT2*∗*6*W by the Chair of the international NAT Committee.

## Figures and Tables

**Table 1:** The allele frequency of *NAT2* polymorphisms among Al-Ahsa population (*n* = 96).

*NAT2* allele	Mutation site	Amino acid change	Reference ID	Acetylation status	Allele frequency (95% CI)
*NAT*2^∗^*4*	—	Wild	—	Fast	0.295 (0.232-0.362)
*NAT*2^∗^*11*	481C>T	Leu161Leu	rs1799929	Fast	0.411 (0.342-0.481)
*NAT*2^∗^*12*	803A>G	Arg268Lys	rs1208	Fast	0.510 (0.44-0.581)
*NAT*2^∗^*13*	282C>T	Tyr94Tyr	rs1041983	Fast	0.354 (0.287-0.422)
*NAT*2^∗^*5*	341T>C	Ile114Thr	rs1801280	Slow	0.427 (0.357-0.497)
*NAT*2^∗^*6*	590G>A	Arg197Gln	rs1799930	Slow	0.292 (0.227-0.356)
*NAT*2^∗^*7*	857G>A	Gly286Glu	rs1799931	Slow	0.057 (0.024-0.09)
*NAT*2^∗^*14*	191G>A	Arg64Gln	rs1801279	Slow	0.010 (-0.0039-0.025)

**Table 2:** The *NAT2* gene representing different genotype frequencies among Al-Ahsa population (*n* = 96).

*NAT2* allele	Wild type	Heterozygote	Homozygote
	Frequency (proportion, 95% CI)	Frequency (proportion, 95% CI)	Frequency (proportion, 95% CI)
*NAT*2^∗^*11* (481C>T)	C/C: 39 (0.406, 0.308-0.504)	C/T: 35 (0.365, 0.268-0.461)	T/T: 22 (0.229, 0.145-0.313)
*NAT*2^∗^*12* (803A>G)	A/A: 28 (0.292, 0.201-0.383)	A/G: 38 (0.396, 0.298-0.494)	G/G: 30 (0.313, 0.22-0.405)
*NAT*2^∗^*13* (282C>T)	C/C: 41 (0.427, 0.328-0.526)	C/T: 42 (0.438, 0.338-0.537)	T/T: 13 (0.135, 0.067-0.204)
*NAT*2^∗^*5* (341T>C)	T/T: 36 (0.375, 0.278-0.472)	T/C: 38 (0.396, 0.298-0.494)	C/C: 22 (0.229, 0.145-0.313)
*NAT*2^∗^*6* (590G>A)	G/G: 52 (0.542, 0.442-0.641)	G/A: 32 (0.333, 0.239-0.428)	A/A: 12 (0.125, 0.059-0.191)
*NAT*2^∗^*7* (857G>A)	G/G: 85 (0.885, 0.822-0.949)	G/A: 11 (0.115, 0.051-0.178)	A/A: 0 (0.00)
*NAT*2^∗^*14* (191G>A)	G/G: 94 (0.979, 0.951-1.008)	G/A: 2 (0.021, -0.0077-0.049)	A/A: 0 (0.00)

**Table 3:** Observed and expected (by using Hardy-Weinberg equilibrium) frequencies of homozygote heterozygote *NAT2* alleles among Al-Ahsa population.

*NAT2* allele		Homozygote *NAT*2^∗^*11*	Heterozygote *NAT*2^∗^*11*	*NAT2* genotype without *NAT*2^∗^*11*
*NAT*2^∗^*11*	Observed number	22	35	39
	Expected number	16.25	46.5	33.3
	*χ* ^2^ of *NAT*2^∗^*11* = 5.87 *χ*^2^ > 3.84 at 1 degree of freedom, *p* value 0.05. Not within Hardy-Weinberg equilibrium.			
*NAT*2^∗^*12*		Homozygote *NAT*2^∗^*12*	Heterozygote *NAT*2^∗^*12*	*NAT2* genotype without *NAT*2^∗^*12*
	Observed number	30	38	28
	Expected number	25	48	23
	*χ* ^2^ of *NAT*2^∗^*12* = 4.15 *χ*^2^ > 3.84 at 1 degree of freedom, *p* value 0.05. Not within Hardy-Weinberg equilibrium.			
*NAT*2^∗^*13*		Homozygote *NAT*2^∗^*13*	Heterozygote *NAT*2^∗^*13*	*NAT2* genotype without *NAT*2^∗^*13*
	Observed number	13	42	41
	Expected number	12	44	40
	*χ* ^2^ of *NAT2*∗*13* = 0.18 *χ*^2^ < 3.84 at 1 degree of freedom, *p* value 0.05. Within Hardy-Weinberg equilibrium.			
*NAT*2^∗^*5*		Homozygote *NAT*2^∗^*5*	Heterozygote *NAT*2^∗^*5*	*NAT2* genotype without *NAT*2^∗^*5*
	Observed number	22	38	36
	Expected number	17.5	47	31.5
	*χ* ^2^ of *NAT*2^∗^*5* = 3.51 *χ*^2^ < 3.84 at 1 degree of freedom, *p* value 0.05. Within Hardy-Weinberg equilibrium.			
*NAT*2^∗^*6*		Homozygote *NAT*2^∗^*6*	Heterozygote *NAT*2^∗^*6*	*NAT2* genotype without *NAT*2^∗^*6*
	Observed number	12	32	52
	Expected number	8.2	39.7	48.2
	*χ* ^2^ of *NAT*2^∗^*6* = 3.59 *χ*^2^ < 3.84 at 1 degree of freedom, *p* value 0.05. Within Hardy-Weinberg equilibrium.			
*NAT*2^∗^*7*		Homozygote *NAT*2^∗^*7*	Heterozygote *NAT*2^∗^*7*	*NAT2* genotype without *NAT*2^∗^*7*
	Observed number	0	11	85
	Expected number	0.32	10.4	85.3
	*χ* ^2^ of *NAT*2^∗^*7* = 0.35 *χ*^2^ < 3.84 at 1 degree of freedom, *p* value 0.05. Within Hardy-Weinberg equilibrium.			
*NAT*2^∗^*14*		Homozygote *NAT*2^∗^*14*	Heterozygote *NAT*2^∗^*14*	*NAT2* genotype without *NAT*2^∗^*14*
	Observed number	0	2	94
	Expected number	0.01	2	94
	*χ* ^2^ of *NAT*2^∗^*14* = 0.01 *χ*^2^ < 3.84 at 1 degree of freedom, *p* value 0.05. Within Hardy-Weinberg equilibrium.			

**Table 4:** Predicted acetylation phenotype among Al-Ahsa population (*n* = 96).

Acetylation phenotype^∗^	Number of samples	Proportion (95%CI^∗∗^)
Fast	5	0.05 (0.01-0.09)
Intermediate	33	0.34 (0.25-0.44)
Slow	58	0.61 (0.51-0.70)
Total	96	

^∗^The acetylation phenotype was determined depending on the trimodal distribution pattern based on online software http://nat2pred.rit.albany.edu/. ^∗∗^CI: confidence interval.

**Table 5:** *NAT2* haplotypes among unrelated Al-Ahsa population (*n* = 96).

Haplotype	Number	Frequency	Predicted phenotype
*NAT*2^∗^*6C*	24	25.00%	Slow
*NAT*2^∗^*5A*	22	22.92%	Slow
*NAT*2^∗^*5*U	12	12.50%	Slow
*NAT*2^∗^*5B*	11	11.46%	Slow
*NAT*2^∗^*5TB* new	7	7.29%	Slow
*NAT*2^∗^*6W* new	2	2.08%	Slow
*NAT*2^∗^*5G*	2	2.08%	Slow
*NAT*2^∗^*5R*	2	2.08%	Slow
*NAT*2^∗^*6A*	2	2.08%	Slow
*NAT*2^∗^*4*	2	2.08%	Fast
*NAT*2^∗^*5AB* new	1	1.04%	Slow
*NAT*2^∗^*5ZA* new	1	1.04%	Slow
*NAT*2^∗^*11A*	1	1.04%	Fast
*NAT*2^∗^*12A*	1	1.04%	Fast
*NAT*2^∗^*12H*	1	1.04%	Fast
*NAT*2^∗^*14B*	1	1.04%	Slow
*NAT*2^∗^*5E*	1	1.04%	Slow
*NAT*2^∗^*5TA*	1	1.04%	Slow
*NAT*2^∗^*6F*	1	1.04%	Slow
*NAT*2^∗^*7C*	1	1.04%	Slow
Total	96	100%	

**Table 6:** Distribution of *NAT2* alleles among Al-Ahsa population compared with various.human population (no.of alleles = 192).

Population	No. alleles	*NAT*2^∗^4	*NAT*2^∗^5	*NAT*2^∗^6	*NAT*2^∗^7	*NAT*2^∗^11	*NAT*2^∗^*1*2	*NAT*2^∗^*1*3
Saudi (current study)	192	0.295	0.427	0.292	0.057	0.411	0.510	0.354
Saudi [12]	974	0.27	0.47	0.24	0.02			
Emiratis [13]	212	0.18	0.54	0.20	0.04			
Emiratis [14]	1110	0.13	0.364	0.129	0.27			
Omanis [15]	254	0.18	0.424	0.30	0.04	0	0.10	0
Jordan [16]	136	0.23	0.485	0.30		0.40	0.30	0.30
Egyptian [17]	400	0.22	0.497	0.26	0.03			
Sudan [18]	254	0.087	0.473	0.28	0.03	0	0.08	0.001
Indians [19]	188	0.10	0.346	0.20	0.05	0	0.30	0
Japan [20]	346	0.66	0.01	0.20	0.13	0	0	0
Chinese	560	0.60	0.136	0.40	0.43	0.1	0	0.6
Korean [21]	2000	0.66	0.016	0.20	0.11	0	0	0
German [22]	1688	0.22	0.46	0.27	0.013	0	0	0.15

## Data Availability

All data are within the manuscript.

## Supplementary Materials

Supplementary Materials Supplementary Table: NAT2 haplotypes among Al-Ahsa population.
