# Correction to “Study on Genotyping Polymorphism and Sequencing of N‐Acetyltransferase 2 (NAT2) Among Al‐Ahsa Population”

**DOI:** 10.1155/bmri/9847214

**Published:** 2026-07-13

M. A. Zahra, M. Kandeel, S. A. Aldossary, A. Al‐Taher, “Study on Genotyping Polymorphism and Sequencing of N‐Acetyltransferase 2 (NAT2) Among Al‐Ahsa Population,” *BioMed Research International*, 2020, 8765347, https://doi.org/10.1155/2020/8765347

In the article, there are discrepancies between the proportions of acetylation phenotype in the abstract and in Table 4, introduced due to rounding errors. The correct Table 4 is presented below:

**Table 4:** Acetylation phenotype distribution among the study population.

Acetylation phenotype ^∗^	Number of samples	Proportion (95% CI ^∗∗^)
Fast	5	5.21% (0.01–0.09)
Intermediate	33	34.38% (0.25–0.44)
Slow	58	60.42% (0.51–0.70)
Total	96	

^∗^The acetylation phenotype was determined depending on the trimodal distribution pattern based on online software http://nat2pred.rit.albany.edu/.

^∗∗^CI: confidence interval.

We apologize for these errors.

