# Supplementary material for: Study on Genotyping Polymorphism and Sequencing of N-Acetyltransferase 2 (NAT2) among Al-Ahsa Population
Source: Biomed Res Int. 2020 Jun 15;2020:8765347. doi: 10.1155/2020/8765347 (PMC7312966; doi:10.1155/2020/8765347)
Supplement: Supplementary Materials — Supplementary Table: NAT2 haplotypes among Al-Ahsa population. [file 8765347.f1.docx]

| **Haplotype** | **G191A** | **C282T** | **T341C** | **C481T** | **G590A** | **A803G** | **G857A** | **Allele frequency** | **Phenotype** |
| --- | --- | --- | --- | --- | --- | --- | --- | --- | --- |
| **NAT2*4** | G | C | T | C | G | A | G | 2.08% | Fast |
| ***(NAT2*5AB) New** | G | C | C | T | G | A | G | 1.04% | Slow |
| **(NAT2*5ZA) New** | A | T | C | T | G | G | G | 1.04% | Slow |
| **NAT2*11A** | G | C | T | T | G | A | G | 1.04% | Fast |
| **NAT2*12A** | G | C | T | C | G | G | G | 1.04% | Fast |
| ****NAT2*12H** | G | C | T | C | G | G | G | 1.04% | Fast |
| **NAT2*14B** | A | T | T | C | G | A | G | 1.04% | Slow |
| **NAT2*5E** | G | C | C | C | A | A | G | 1.04% | Slow |
| **NAT2*5TA** | G | T | C | C | G | G | A | 1.04% | Slow |
| **NAT2*6A** | G | T | T | C | A | A | G | 2.08% | Slow |
| **NAT2*6F** | G | C | T | C | A | G | G | 1.04% | Slow |
| **NAT2*7C** | G | T | T | C | G | G | A | 1.04% | Slow |
| **(NAT2*6W) New** | G | T | T | C | A | G | A | 2.08% | Slow |
| **NAT2*5G** | G | T | C | T |  | G | A | 2.08% | Slow |
| **NAT2*5R** | G | T | C | C | A | G | G | 2.08% | Slow |
| **(NAT2*5TB) New** | G | T | C | T | G | G | A | 7.29% | Slow |
| **NAT2*5B** | G | C | C | T | G | G | G | 11.46% | Slow |
| **NAT2*5U** | G | T | C | T | A | G | G | 12.50% | Slow |
| **NAT2*5A** | G | C | C | T | G | A | G | 22.92% | Slow |
| **NAT2*6C** | G | T | T | C | A | G | G | 25.00% | Slow |

**Table:** NAT2 Haplotypes among Al-Ahsa population.
